# Astragaloside-IV promotes autophagy via the Akt/mTOR pathway to improve cellular lipid deposition

**DOI:** 10.1097/MD.0000000000037846

**Published:** 2024-04-19

**Authors:** Guo Liu, Ye-Hui Wang, Ting Zhang, Ya-Qiong Li, Xin-Yue Chen, Wei Dong, Wei Li, Qi-Xiang Miao, Wen-Bo Qiao, Hui-Qiang Tian, Shi-Long Yin

**Affiliations:** a Qionglai Hospital of Traditional Chinese Medicine, Qionglai, Chengdu, Sichuan, China; b Sichuan Province Orthopedic Hospital, Chengdu, Sichuan, China; c Hospital of Chengdu University of Traditional Chinese Medicine, Chengdu, Sichuan, China.

**Keywords:** Akt/mTOR, astragaloside-IV, autophagy, fatty liver

## Abstract

The current study aimed to investigate the potential role of astragaloside IV (AS-IV) in improving cellular lipid deposition and its underlying mechanism. A fatty liver cell model was established by treating hepatoma cells with palmitic acid. AS-IV and SC79 were used for treatment. Oil Red O staining was applied to detect intracellular lipid deposition, and transmission electron microscopy was utilized to assess autophagosome formation. Immunofluorescence double staining was applied to determine microtubule-associated proteins 1A/1B light chain 3 (LC3) expression. Western blot analysis was performed to detect the expression of LC3, prostacyclin, Beclin-1, V-akt murine thymoma viral oncogene homolog (Akt), phosphorylated Akt, mTOR, and phosphorylated mTOR. Oil Red O staining revealed that AS-IV reduced intracellular lipid accumulation. Further, it increased autophagosome synthesis and the expression of autophagy proteins LC3 and Beclin-1 in the cells. It also reduced the phosphorylation levels of Akt and mTOR and the levels of prostacyclin. However, the effects of AS-IV decreased with SC79 treatment. In addition, LC3B + BODIPY493/503 fluorescence double staining showed that AS-IV reduced intracellular lipid deposition levels by enhancing autophagy. AS-IV can reduce lipid aggregation in fatty liver cells, which can be related to enhanced hepatocyte autophagy by inhibiting the Akt/mTOR signaling pathway.

## 1. Introduction

Nonalcoholic fatty liver disease (NAFLD) refers to a clinicopathological syndrome characterized by excessive accumulation of fat in hepatocytes caused by alcohol and other definite liver-damaging factors, and acquired metabolic stress liver injury that is closely related to insulin resistance and genetic predisposition, including nonalcoholic fatty liver disease or simple steatosis, nonalcoholic steatohepatitis, liver fibrosis, and liver cirrhosis. Eventually, hepatocellular carcinoma can develop without significant cirrhosis.^[1,2]^ In recent years, the incidence and prevalence of NAFLD have increased rapidly worldwide, and are increasing annually among younger individuals.^[3,4]^ However, there is no specific drug for preventing and treating nonalcoholic fatty liver disease. At present, the drugs used in clinical treatment for NAFLD include lipid-lowering drugs, peroxisome proliferator-activated receptor agonists, and hepatoprotective and anti-inflammatory drugs. However, most of them are not significantly effective and have remarkable side effects. Moreover, the US Food and Drug Administration did not approve any specific drug for treating NAFLD.^[5]^ Therefore, a safe and effective novel therapeutic drug for NAFLD should be explored. Natural herbal medicines are characterized by complex compositions and have multiple targets. Thus, safer and more effective drugs for treating nonalcoholic fats can be developed.

Autophagy, a core molecular pathway for the preservation of cellular and organismal homeostasis, is the principal mechanism that mediates the delivery of various cellular cargoes to lysosomes for degradation and recycling.^[6,7]^ In 2009, lipophagy, another specific type of autophagy, was first discovered.^[8]^ Subsequently, this autophagy-lysosome-dependent degradation pathway of lipid droplets (LDs) has been found in different cells, and it plays an important role in maintaining lipid metabolic homeostasis in the liver.^[9,10]^ In addition to being hydrolyzed to free fatty acids by specific lipases, triglycerides (TG) stored in LDs in hepatocytes can be selectively degraded by lipid autophagy to provide energy if the body is nutrient-deficient.^[9]^ Therefore, autophagy is closely related to nonalcoholic fatty liver disease.

Astragalus membranaceus is one of the commonly used traditional Chinese medicines in Bigu therapy in traditional Chinese medicine. It is widely utilized as an alternative treatment for immunological diseases, metabolic diseases, and liver fibrosis.^[11–13]^ Astragaloside IV (AS-IV), the main active substance of *Astragalus membranaceus*, with the molecular formula of C_41_H_68_O_14_, can inhibit the abnormal expression of lipid synthesis-related genes, improve the ability to resist oxidative stress, reduce the degree of endoplasmic reticulum stress, decrease inflammatory response, and inhibit apoptosis.^[14–16]^ AS-IV can improve liver lipid deposition in mice with NAFLD.^[17,18]^ Further, it can have biological effects by acting on the Akt/mTOR signaling pathway.^[19]^ In addition, autophagy is closely related to the Akt/mTOR signaling pathway. Activation of this pathway can regulate cell growth and metabolism, inhibit activity of autophagy-related proteins, and reduce autophagy, which have implications for cell growth, energy metabolism, and pathogenesis of several diseases.^[20–22]^ Hence, AS-IV can enhance autophagy and improve hepatocyte lipid deposition by inhibiting the Akt/mTOR signaling pathway, which may be another mechanism of Astragalus in treating nonalcoholic liver.

## 2. Materials and methods

### 2.1. Reagents

The HepG2 cells (CL-0103) were supplied by Procell (Wuhan, China). AS-IV (84687-43-4) was obtained from Xi’an Tian Guangyuan Biological Technology Co., Ltd. (Xi’an, China). Palmitic acid (SP8060) was purchased from Shanghai Yuanpei Biotechnology Co., Ltd. The BCA Protein Assay Kit (P0009) was purchased from Beyotime Biotech Inc. (Shanghai, China). Antibodies against V-akt murine thymoma viral oncogene homolog (Akt) (A17909), Beclin-1 (A7353), microtubule-associated proteins 1A/1B light chain 3 (LC3) (A19665), mTOR (A2445), prostacyclin (p62) (A7758), phosphorylated Akt (p-Akt) (AP0637), phosphorylated mTOR (p-mTOR) (AP0094), and β-actin (AC026) were supplied by ABclonal. Secondary antibodies against goat anti-rabbit IgG (H&L) antibody (Biotin) (S0001) were purchased from Affinity (Jiangsu, China). Cy3-AffiniPure Goat Anti-Rabbit IgG(H + L) (GB21303) and 4 ′,6-diamidino-2-phenylindole staining reagent (G1012) were provided by Servicebio (Wuhan, China). TP kits (A045-2-1), TC kits (A111-2-1), and TG kits (A110-2-1) were supplied by Nanjing Institute of Biological Engineering.

### 2.2. CCK-8 assay

After 48 hours of treatment, the supernatant was aspirated and discarded. The Cell Counting Kit-8 (CCK-8) reagent was diluted at a ratio of 1:10 in serum-free medium, and 400 μL/well of the diluted CCK-8 working solution was added to the 6-well plate. The culture plate was gently shaken several times, and the culture was continued at 37 °C and 5% CO_2_ for 2 hours. The absorbance values of each well were measured at a wavelength of 450 nm using a microplate reader.

### 2.3. Oil Red O staining

After drug treatment, the cell culture medium was removed and the cells were washed twice with phosphate buffered saline (PBS). Oil Red O fixative was added and fixed for 20 minutes. Then, the fixative was discarded and washed twice with distilled water. Next, 60% isopropanol was added and washed for 20 to 30 seconds. Then, the staining solution was discarded, rinsed with 60% isopropanol for 20 to 30 seconds until the stroma was clear, and washed with water for 2 to 5 times. Mayer hematoxylin staining solution was added, and the nuclei were counterstained for 1 to 2 minutes. The staining solution was discarded and then washed with water for 2 to 5 times. Oil Red O buffer was added for 1 minute and discarded, and distilled water was added to cover the cells, observed under a microscope, and photographed.

### 2.4. Determination of TG and TC

The cells were lysed using the TG and total cholesterol (TC) lysates. The bicinchoninic acid assay was used to determine the cellular protein concentration. The working solution for determining TG or TC content was prepared at a ratio of 4:1. Then, 190 μL of the working solution was sucked in the microplate with a pipettes gun, and 10 μL of the standard sample and ethanol was sucked (blank control reaction). After the reaction in an incubator with a temperature of 37 °C for 10 minutes, the blank tube should be initially set to zero, and the OD570 value should be detected at 570 nm by a microplate reader. The data should be recorded. After the experiment, the TG or TC content should be corrected by per milligram of protein concentration.

### 2.5. Immunofluorescent double staining

The cell climbing slices were placed into the staining jar and washed with PBS for 3 times, 5 minutes each time. Then, the membrane-breaking solution was added to cover the cells, and incubated at room temperature for 10 minutes. The solution was blocked with bovine serum, and the primary antibody LC3 was added and incubated at 4 °C overnight. After washing with PBS, the secondary antibody CY3-labeled goat anti-rabbit was added. Next, BODIPY 493/503 staining solution was added. The solution was covered with foil to prevent exposure to light, and 4′,6-diamidino-2-phenylindole was added for incubation at room temperature. Finally, the slices were sealed with anti-fluorescence attenuation sealing agent. A scanning software was used to capture images of the slices, initially assess the whole tissue under low magnification, and then capture 200 microimages individually, with a 1 field of view.

### 2.6. Transmission electron microscopy

The cell samples were prefixed with 3% glutaraldehyde, postfixed with 1% osmium tetroxide, dehydrated with acetone, embedded in Epon812, and then cut into ultrathin sections using an ultramicrotome. Next, the sections were mounted and transferred to 200-mesh copper grids. The grids were initially stained with uranyl acetate in the dark for 10 to 15 minutes, followed by lead citrate staining in a carbon dioxide-free environment for 1 to 10 minutes. After staining at room temperature, the grids were washed 3 times with ultrapure water, gently dried with a filter paper, placed in copper grid boxes, and left to dry overnight at room temperature. Subsequently, the JEM-1400FLASH transmission electron microscope was used to capture and analyze copper grid images.

### 2.7. Western blot analysis

The protein levels of Akt, p-Akt, mTOR, p-mTOR, Beclin-1, p62, and LC3-II/I in HepG2 cells were detected via Western blot analysis. Total cellular proteins were extracted using radioimmunoprecipitation assay buffer lysates. The proteins were transferred to the polyvinylidene difluoride membrane using the wet rotation method and blocked at room temperature for 1 hour. The primary antibody was incubated overnight, and the membrane was washed 3 times with Tris-buffered saline with 0.1% Tween® 20 detergent for 5 minutes each time. The secondary antibody was incubated at room temperature for 2 hours, and the membrane was washed 3 times with Tris-buffered saline with 0.1% Tween® 20 detergent for 10 minutes each time. Then, the ECL developer solution was uniformly added to the membrane for exposure development. The bands were scanned by exposure using the Tianneng GIS chassis control software version 2.0, and the results were expressed as the relative expression of the target protein.

### 2.8. Statistical analyses

The Statistical Package for the Social Sciences software version 26 (IBM Inc.) was used to analyze the data generated in the charts in this experiment. Figures were made using GraphPad Prism version 9.5.1 (GraphPad Software, Inc.). All data were presented as mean ± standard error of the mean, and all experiments were performed 3 times. Differences among groups were analyzed using one-way analysis of variance, followed by the Fisher post hoc test. Between-group differences were statistically analyzed using the unpaired, two-tailed Student *t* test. A *P* value of <.05 indicated statistically significant difference.

## 3. Results

### 3.1. Effect of AS-IV on lipid concentrations in HepG2 cells

**Figure 1.**
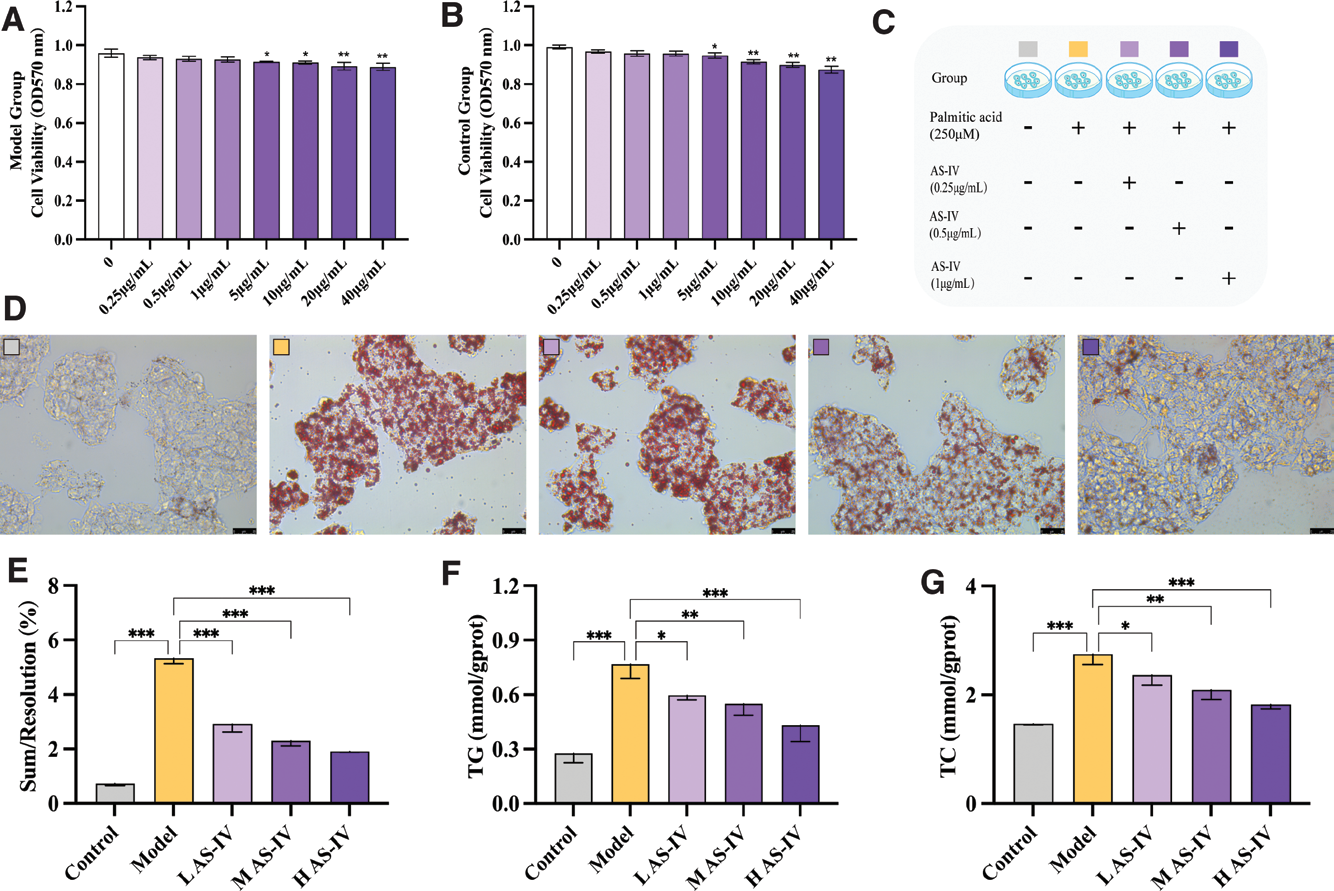
Screening of the effect of AS-IV at different concentrations on lipid concentrations in HepG2 cells. (A, B) Quantitative analysis of the cell viability rate. (C) Schematic representation of cell grouping and pretreatment. (D) Representative images of Oil Red O staining of intracellular lipid droplets in each group. (E) Quantitative analysis of Oil Red O staining. (F,G) Quantitative analysis of the TG and TC levels. Data were presented as mean ± standard deviations (n = 3). *P < .05, **P < .01, ***P < .001, ****P < .0001, nsp > 0.05.

The effect of HepG2 cell treatment with palmitic acid at a concentration of 250 μM and AS-IV at different concentrations (0.25, 0.5, 1, 5, 10, 20, and 40 μg/mL) on cell proliferation was assessed using the CCK-8 assay. After 24 hours of treatment with AS-IV at different concentrations, regardless of palmitic acid treatment, the cell viability decreased in a dose-dependent manner. The cell proliferation activity significantly decreased with treatment with AS-IV at concentrations of 5, 10, 20, or 40 μg/mL (*P* < .05). The safe dose range of AS-IV for HepG2 cells was 0 to 1 μg/mL. Therefore, the low doses of AS-IV do not affect HepG2 cell proliferation activity. This is consistent with the results of the study of Wang et al^[23]^ Therefore, AS-IV at concentrations of 0 to 1 μg/mL was used for subsequent experiments. See Figure 1A, B.

The number of intracellular LDs and the TC and TG levels in the model group increased significantly compared with those of the control group (*P* < .01). Thus, palmitic acid can induce intracellular lipid deposition of HepG2. After treatment with AS-IV at different concentrations, the intracellular LD number and the TC and TG levels gradually decreased in the palmitic acid-treated cells (*P* < .05). Hence, AS-IV has lipid-lowering effects. Furthermore, high-dose AS-IV had the best lipid-lowering effect (*P* < .01). See Figure 1C–F.

### 3.2. Effect of AS-IV on the autophagy level in HepG2 cells

**Figure 2.**
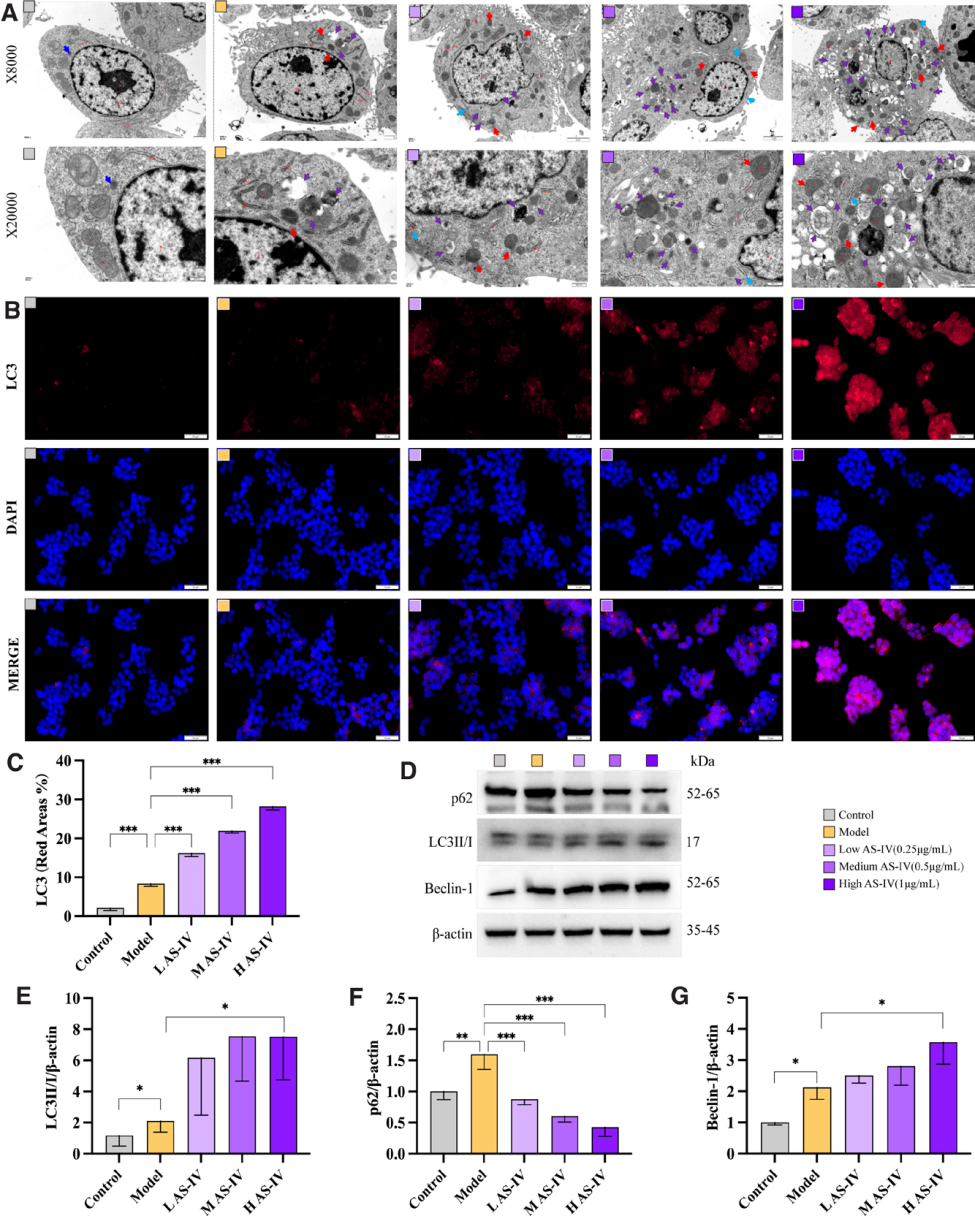
Effect of AS-IV on the autophagy level in HepG2 cells. (A) Representative transmission electron microscopy images of HepG2 cells in each group. The red arrow shows mild pyknosis of the mitochondria. The blue arrow shows mild expansion of the rough endoplasmic reticulum, and the purple arrow shows autophagy. (B) Representative images of immunofluorescence staining of LC3 in each group. (C) Quantitative analysis of immunofluorescence staining. (D) Bands of autophagy-related indicators, including LC3II/I, Beclin-1, and p62. (E–G) Quantitative analysis of Western blotting findings. Data were presented as means ± standard deviations (n = 3). *P < .05, **P < .01, ***P < .001, ****P < .0001, nsp > 0.05.

The HepG2 cell mitochondria exhibited a normal elliptical morphology with closely arranged cristae, clear and straight outlines, and uniform gray electron density in the control group. The rough endoplasmic reticulum structure appeared normal and vesicular, with a small amount of lysosomes visible in the cytoplasm. In comparison to the control group, the model group cells had significant mitochondrial condensation, reduced or absent cristae, widened intercristal spaces, increased membrane density, deepened electron density, and a low concentration of autophagosomes and LDs in the cytoplasm. After AS-IV treatment, the cells had mild mitochondrial condensation with reduced size, decreased or absent cristae, widened intercristal spaces, increased membrane density, deepened electron density, and the presence of autophagosomes in the cytoplasm. Notably, the high-dose AS-IV group exhibited a significant increase in autophagy level compared with the model group. Therefore, AS-IV can promote palmitic acid-induced autophagy in HepG2 cells. See Figure 2A.

Immunofluorescence staining was performed to evaluate the expression of autophagy marker protein LC3 in HepG2 cells. The positive expression rate of LC3 in the cells in the model group significantly increased compared with that in the control group (*P* < .01). After treatment with AS-IV at different concentrations, the expression of LC3 in the cells further increased. The expression of LC3 in the cells treated with AS-IV at low, medium, and high doses significantly increased compared with that in the model group cells (*P* < .01, see Figure 2B, C).

The protein LC3 is a marker of autophagy, and the LC3-II expression directly reflects the number of autophagosomes. These findings combined with those of a study on autophagic substrate p62 were used to analyze the dynamic process of autophagic flux. Western blot analysis showed that compared with the control group, the expression of LC3II/I, beclin-1, and p62 in the model group cells increased moderately (*P* < .05). Thus, palmitic acid can induce autophagic flux blockade in HepG2 cells and abnormal accumulation of autophagosomes and autophagic substrates. Palmitic acid-induced HepG2 cells treated with AS-IV at different concentrations had a concentration-dependent increase in the expression of LC3II/I and beclin-1, and a concentration-dependent decrease in the expression of p62. The high-dose AS-IV group showed statistical significance compared with the model group (*P* < .05). Hence, AS-IV can significantly enhance the upstream stage of autophagic flux (LC3-II) in HepG2 cells and improve palmitic acid-induced autophagic flux blockade (p62), thereby inducing lipophagy and promoting the degradation of excessive LDs caused by palmitic acid. See Figure 2D–G.

### 3.3. Effect of AS-IV on the Akt/mTOR signaling pathway in HepG2 cells

**Figure 3.**
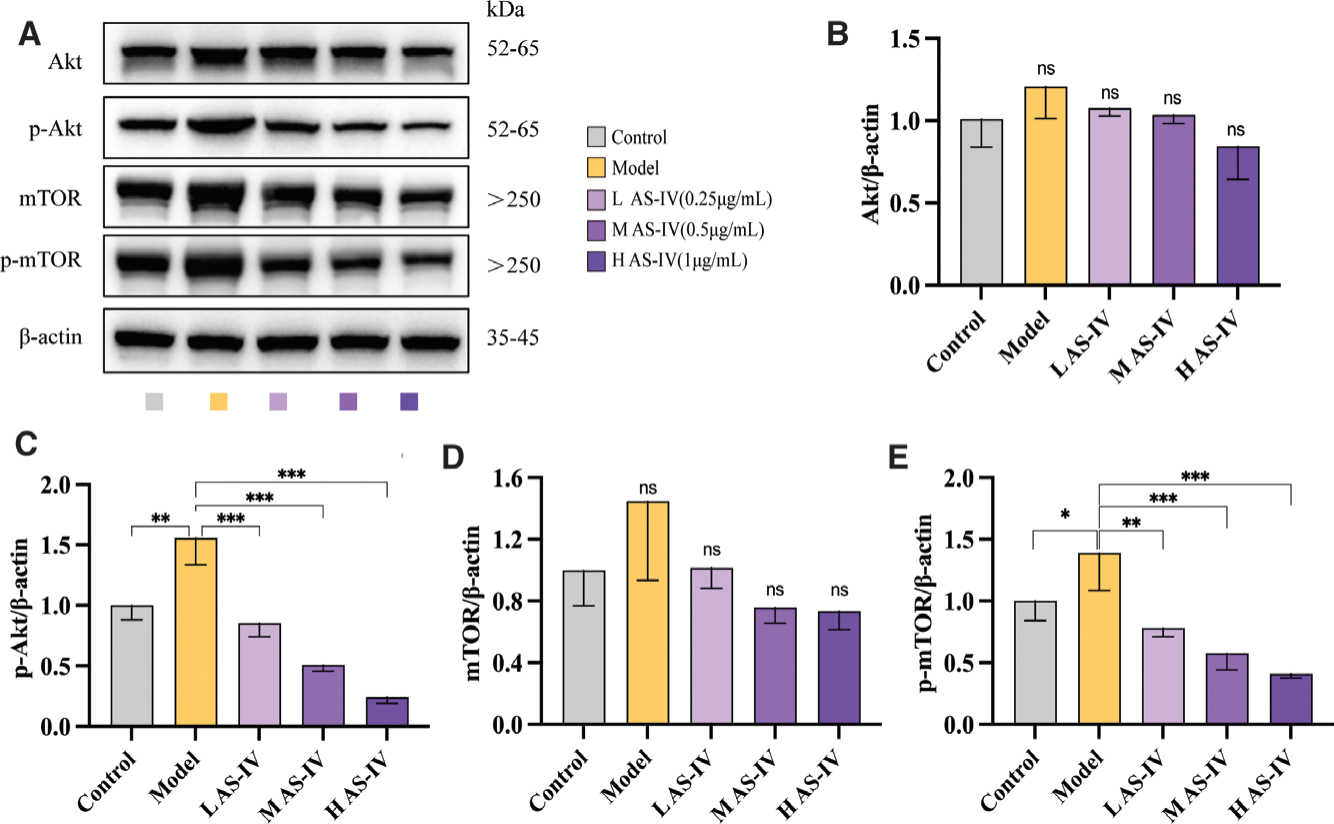
Effect of AS-IV on the Akt/mTOR signaling pathway in HepG2 cells. (A) Western blot analysis of Akt, p-Akt, mTOR, and p-mTOR. (B–E) Quantitative analysis of Western blot analysis results. Data were presented as mean ± standard deviation (n = 3). *P < .05, **P < .01, ***P < .001, ****P < .0001, nsp > 0.05.

As shown in Figure 3, the expression of intracellular Akt and mTOR in the model group did not significantly change compared with that in the control group (*P* > .05), and the expression of p-Akt and p-mTOR was significantly upregulated (*P* < .05). The expression of intracellular p-Akt in the low-, medium- and high-dose AS-IV group was significantly upregulated compared with that in the model group (*P* < .01). The expression of intracellular p-mTOR was significantly downregulated in the medium- and high-dose AS-IV group (*P* < .05).

**Figure 4.**
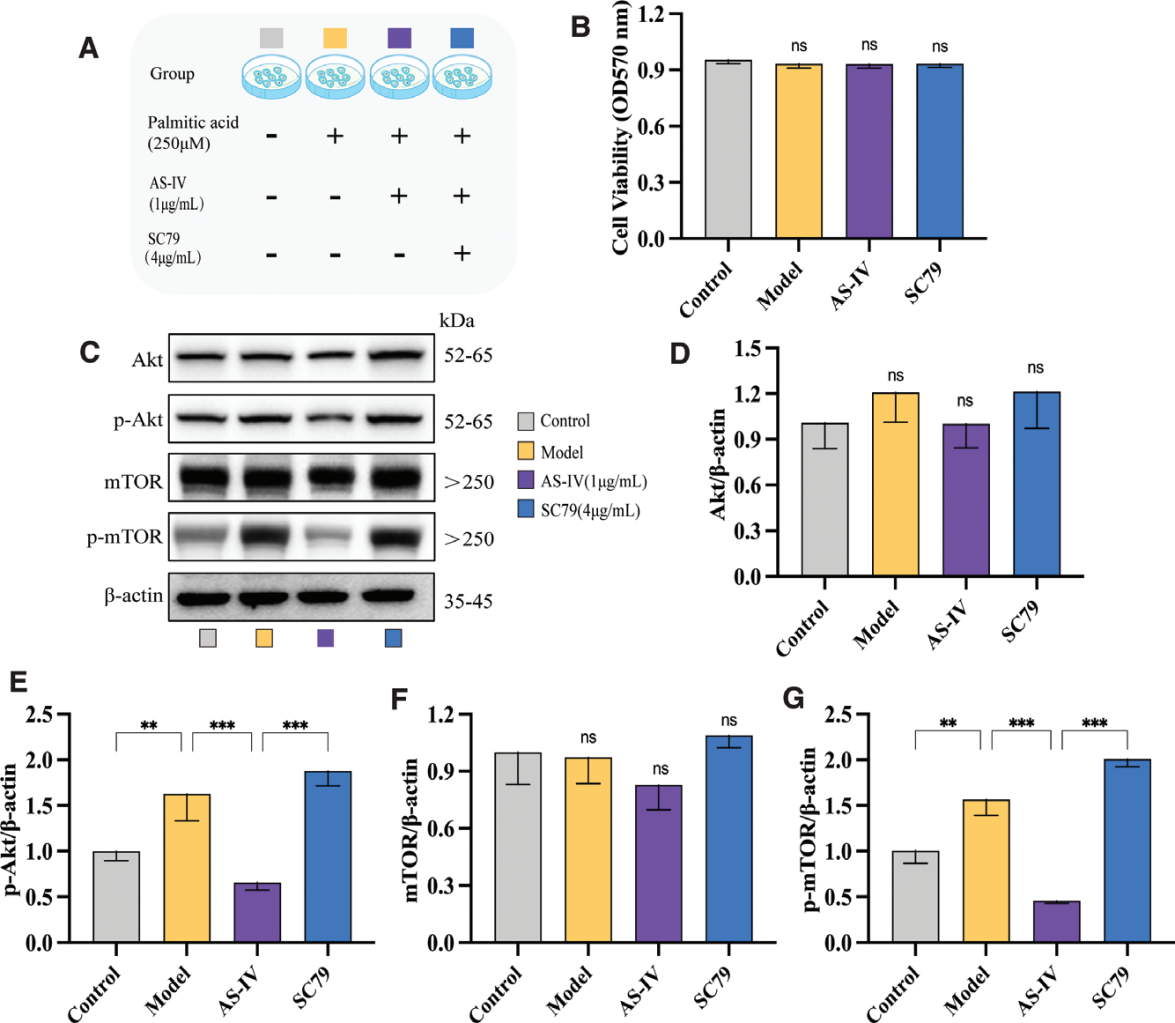
Effect of SC79 on Akt/mTOR induced by AS-IV. (A) Schematic representation of cell grouping and pretreatment. (B) Quantitative analysis of the cell viability rate. (C) Western blot analysis of Akt, p-Akt, mTOR, and p-mTOR. (D–G) Quantitative analysis of Western blot analysis results. Data were presented as mean ± standard deviation (n = 3). *P < .05, **P < .01, ***P < .001, ****P < .0001, nsp > 0.05.

Next, SC79 was used to further validate the effect of AS-IV on the Akt/mTOR signaling pathway. The schematic representation of cell grouping and pretreatment was shown in Figure 4A. The CCK-8 assay showed that the doses of AS-IV and SC79, an agonist of the Akt/mTOR pathway, used in this study had no significant cytotoxic effects on HepG2 cells (Fig. 4B). HepG2 cells were treated with 250 μM of palmitic acid and 1 μg/mL of AS-IV and intervened with 4 μg/mL of SC79. The expression of intracellular p-Akt and p-mTOR was significantly upregulated after palmitic acid treatment (*P* < .05). Meanwhile, the expression of p-Akt and p-mTOR was significantly downregulated after AS-IV treatment (*P* < .01). However, the expression of p-Akt and p-mTOR was significantly upregulated after the addition of SC79 (*P* < .01) (Fig. 4C–G). In conclusion, AS-IV promotes autophagy to alleviate palmitate-induced lipid accumulation in hepG2 cells possibly by inhibiting Akt/mTOR pathway activation.

### 3.4. Effect of SC79 on the autophagy regulation by AS-IV

**Figure 5.**
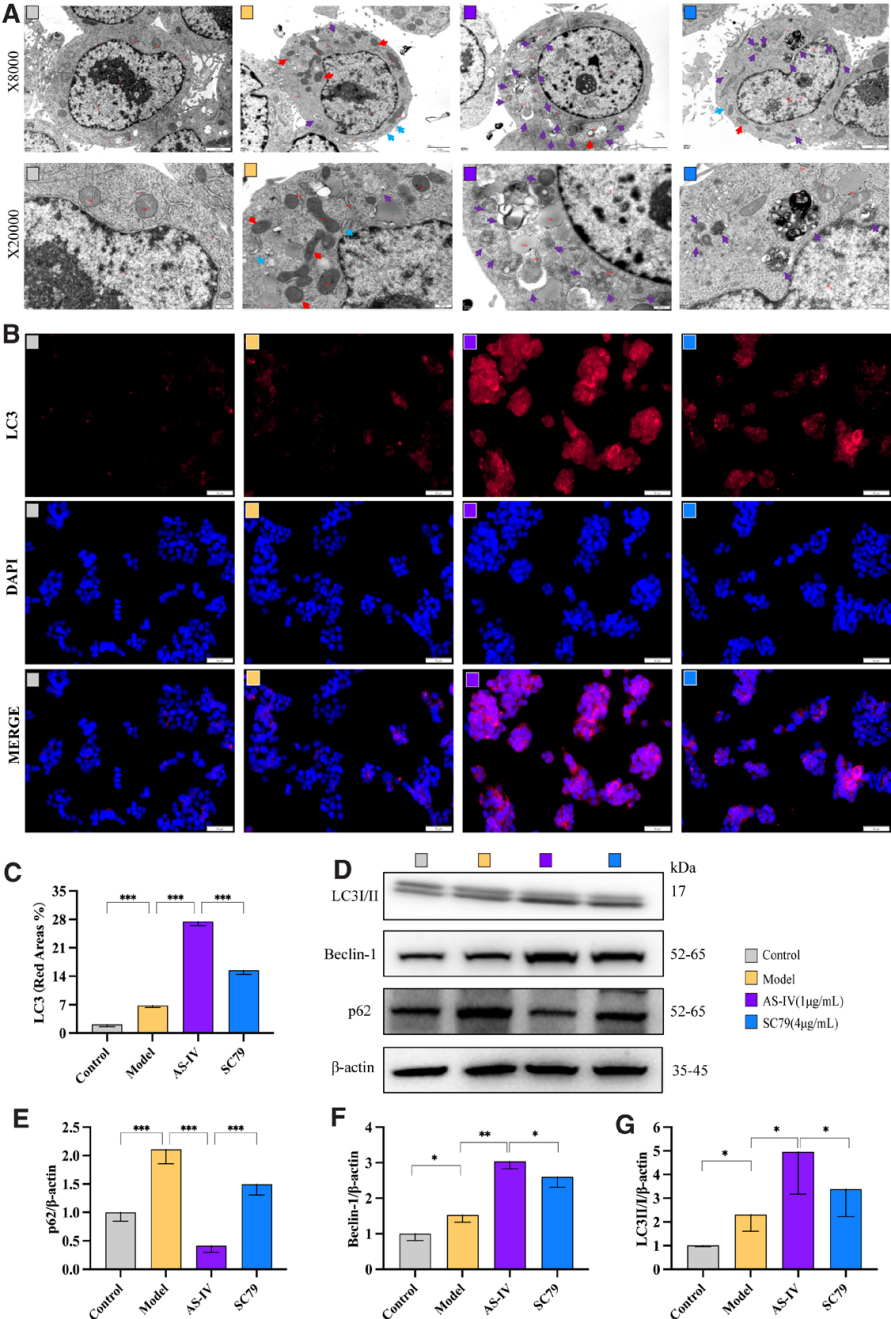
The effect of SC79 on the regulation of autophagy by AS-IV. (A) Representative transmission electron microscopy images of HepG2 cells in each group. The red arrow shows mild pyknosis of mitochondria, the blue arrow shows mild expansion of rough endoplasmic reticulum, and the purple arrow shows autophagy. (B) Representative images of immunofluorescence staining of LC3 in each group. (C) Quantitative analysis of immunofluorescence staining. (D) Bands of autophagy-related indicators, including LC3II/I, Beclin-1, and p62. (E–G) Quantitative analysis of Western blotting findings. Data are presented as the means ± standard deviations (n = 3). *P < .05, **P < .01, ***P < .001, ****P < .0001, nsp > 0.05.

After treating HepG2 cells with palmitic acid, there was significant mitochondrial condensation, reduced or disappeared cristae, widened intermembrane space, increased membrane density, deepened electron density, and a small amount of autophagy and LDs in the cytoplasm. Treatment with AS-IV after palmitic acid induction deepened the degree of autophagy in the cytoplasm. Individual mitochondria in the Akt/mTOR pathway activator group showed mild condensation, reduced or disappeared cristae, and mild expansion of the rough endoplasmic reticulum in a vesicular pattern, with reduced degree of cytoplasmic autophagy, compared with that in the AS-IV group. See Figure 5A.

Next, immunofluorescence staining was used to detect the expression of the autophagy marker protein LC3. The expression of LC3 in the model group significantly increased compared with that in the control group (*P* < .01). After AS-IV intervention, the expression of LC3 in the cells further increased (*P* < .01). Meanwhile, treatment with SC79 reversed the promoting effect of AS-IV on LC3 expression (*P* < .01). The SC79 inhibited the promoting effect of AS-IV on autophagosome formation. Therefore, AS-IV can induce autophagy in HepG2 cells by inhibiting the activation of the Akt/mTOR pathway. See Figure 5B and C.

Western blot analysis was used to detect the expression of autophagy-related proteins LC3I, LC3II, p62, and beclin-1. The expression of LC3II/I, beclin-1, and p62 in the model group cell significantly increased compared with that in the control group (*P* < .05). The expression of LC3II/I and beclin-1 in the AS-IV group increased compared with that in the model group (*P* < .05), and the expression of p62 decreased (*P* < .01). The expression of LC3II/I and beclin-1 in the SC79 group decreased compared with that in the AS-IV group (*P* < .05), and the expression of p62 increased (*P* < .01). In summary, AS-IV enhances autophagy in HepG2 cells and alleviates palmitic acid-induced autophagic flux blockade, a process related to the inhibition of Akt/mTOR signaling pathway activation. See Figure 5D–G.

### 3.5. Effect of SC79 on AS-IV-regulated autophagy improvement of palmitic acid-induced lipid accumulation

**Figure 6.**
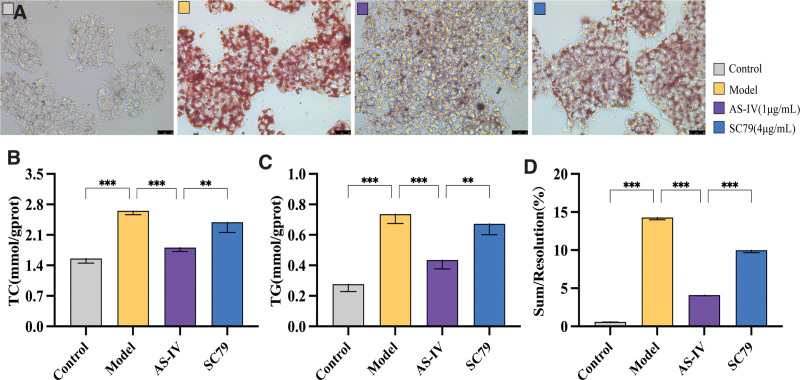
Effect of SC79 on AS-IV-regulated autophagy improvement of palmitic acid-induced lipid accumulation. (A) Representative images of Oil Red O staining of intracellular lipid droplets in each group. (B, C) Quantitative analysis of the TG and TC levels. (D) Quantitative analysis of Oil Red O staining. Data were presented as means ± standard deviations (n = 3). *P < .05, **P < .01, ***P < .001, ****P < .0001, nsp > 0.05.

The number of LDs and the levels of TC and TG in the model group cells significantly increased compared with those in the control group (*P* < .01). However, after treatment with AS-IV, the number of LDs and the levels of TC and TG in the cells significantly decreased (*P* < .01). SC79 reversed the inhibitory effect of AS-IV on cellular lipid deposition (*P* < .01).Therefore, AS-IV enhances autophagy by inhibiting the Akt/mTOR signaling pathway to inhibit palmitic acid-induced lipid accumulation in HepG2 cells. See Figure 6.

**Figure 7.**
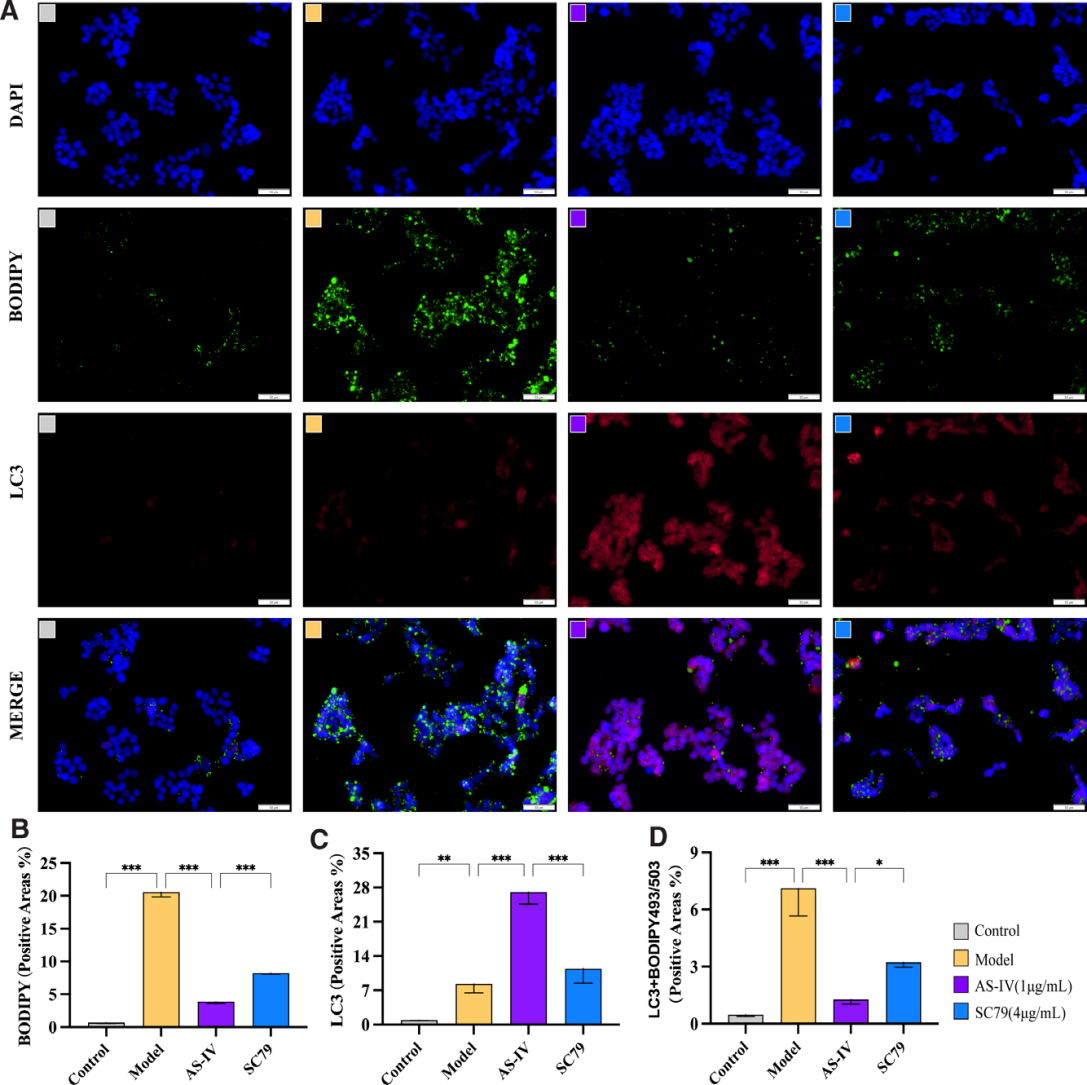
Effect of SC79 on LC3 + BODIPY493/503 expression in HepG2 cells treated with AS-IV. (A) Representative images of immunofluorescence staining of LC3 and lipid droplets in each group. (B–D) Quantitative analysis of immunofluorescence staining findings. Data were presented as means ± standard deviations (n = 3). *P < .05, **P < .01, ***P < .001, ****P < .0001, nsp > 0.05.

Compared with the control group, the green fluorescence (BODIPY) and red fluorescence (LC3) in the palmitic acid group were enhanced simultaneously, and the colocalization was good. Hence, palmitic acid induced the simultaneous increase in LDs and autophagosomes, and a large number of LDs were encapsulated by autophagosomes. After the addition of AS-IV, the green fluorescence (BODIPY) significantly reduced, and the colocalization of BODIPY and LC3 was weakened, which was attributed to the fact that AS-IV promoted autophagy and inhibited the inhibition of palmitic acid on autophagy flow. The autophagy flow was smooth, and a large number of autophagosomes encapsulating LDs were degraded via lipid phagocytosis, resulting in a decreased number of LDs and reduced colocalization. After SC79 treatment, the activation effect of AS-IV on autophagy was significantly weakened, and the green fluorescence (BODIPY) was enhanced again. Moreover, its colocalization with autophagosomes increased again. Thus, AS-IV activates autophagy via the Akt/mTOR signaling pathway, thereby degrading excess lipids in cells. See Figure 7.

## 4. Discussion

NAFLD is strongly associated with metabolic dysfunction.^[24]^ It is an independent factor influencing the pathogenesis of cardiovascular disease.^[25,26]^ Therefore, the prevention and treatment of nonalcoholic fatty liver disease can be beneficial for preventing and treating metabolic and cardiovascular diseases.^[27,28]^ Since the pathogenesis of NASH involves multiple molecular mechanisms, effective prevention and treatment strategies should target multiple pathological pathways and protect related systems or target organs. Astragalus, as a major alternative treatment herb, plays an important role in immune system diseases, respiratory diseases, endocrine system diseases, metabolic diseases, tumors and so on.^[29–32]^

Previous studies have shown that AS-IV can improve endoplasmic reticulum stress in hepatocytes and prevent hepatocyte apoptosis.^[33,34]^ Zhou et al found that AS-IV can reduce fat aggregation in hepatocytes via the AMPK pathway.^[14]^ However, the mechanism has not yet been completely elucidated. Lipid autophagy, another type of autophagy, evidently affects the storage and use of lipids within hepatocytes, and it is closely related to NAFLD and hepatic TG accumulation.^[35]^

Currently, the process of autophagy formation also involves multiple protein pathways, and recent studies have shown that the AMPK/mTOR pathway plays a positive and negative role in autophagy.^[36,37]^ Among them, the AMPK/mTORC1/Unc-51-like autophagy-activating kinase 1 (ULK1)/ATG13 signaling pathway is a cellular energy receptor, and its effect on autophagy has attracted significant attention. The AMPK/mTORC1 pathway is the main regulatory domain of the PI3K/Akt signaling pathway,^[38]^ and the PI3K/Akt/AMPK/mTORC1/ULK1/ATG13 signaling pathway plays a role in autophagy. The downstream substrate protein of AMPK is mainly the mammalian target of rapamycin mTOR, which is a member of the phosphatidylinositol kinase-related kinase family. mTORC1 inhibits autophagy by phosphorylating ULK1 and autophagy-related protein 13.^[39]^ Recent studies have revealed that mTOR in the liver cells of rats with fatty liver is activated to inhibit hepatic autophagy, thereby promoting oxidative stress and fatty acid synthesis.^[40]^ Oxidative stress can promote autophagy, which, in turn, can affect lipogenesis via ERS and LD hydrolysis, degrade intracellular LDs, reduce oxidative stress, and relieve associated liver injury.^[41,42]^ Similar results have been found in animal studies where mTOR inhibition (e.g., rapamycin and its analogues) alleviates NAFLD.^[43]^ Thus, in addition to promoting hepatic lipogenesis, mTORC1 plays an important role in regulating autophagic activity.^[44]^ Therefore, we hypothesized that AS-IV can promote autophagy by inhibiting the Akt/mTOR signaling pathway. The results of Oil Red O-, TC-, and TG-related experiments showed that AS-IV could decrease lipid accumulation induced by palmitic acid. This result was consistent with that of previous studies. Further, the results of transmission electron microscopy, LC3 immunofluorescence staining, and Western blot analysis showed that AS-IV could enhance autophagy and relieve autophagic flux inhibited by palmitic acid.

This study showed that palmitic acid and AS-IV had an effect on the autophagic flow of HepG2 cells, which was attributed to increased compensatory autophagy in cells after palmitic acid stimulation. However, this spontaneous autophagy was not sufficient to completely degrade the excessive LDs. The cells were still in an unfavorable state. AS-IV promoted LD degradation by further enhancing autophagy, which alleviated the lipid accumulation of HepG2 cells. Considering that palmitic acid-induced increase in autophagosomes is accompanied by LD accumulation, we further hypothesized that this may be caused by the blockade of autophagic flux, thereby inhibiting the degradation of LD-containing autophagosomes. Therefore, AS-IV can alleviate lipid accumulation in HepG2 cells by enhancing autophagy and making autophagic flow smoother.

The Akt/mTOR signaling pathway is closely related to autophagy regulation. Therefore, Western blot analysis was performed to detect the expression of Akt/mTOR signaling pathway-related proteins Akt, p-Akt, mTOR, and p-mTOR. AS-IV could inhibit the activation of the Akt/mTOR signaling pathway. Next, by staining LDs with BODIPY while incubating with fluorescent secondary antibodies to specifically visualize the location of LC3, the role of AS-IV was validated via colocalization between LDs and LC3 to reduce intracellular lipid accumulation by enhancing autophagy levels. Finally, the Akt/mTOR signaling pathway agonist SC79 was used to further confirm the effect of AS-IV in increasing the level of autophagy and reducing intracellular lipid accumulation by inhibiting Akt/mTOR.

## 5. Conclusion

AS-IV can reduce lipid aggregation in fatty liver cells, which may be related to the enhancement of hepatocyte autophagy by inhibiting the Akt/mTOR signaling pathway.

## Acknowledgments

Thank professor Wang for writing-review and editing. Also, to the participants for their contributions in engagement as well as feedback. Thank Bullet Edits Limited for the linguistic editing and proofreading of the manuscript.

## Author contributions

**Conceptualization:** Guo Liu, Ye-Hui Wang.

**Data curation:** Ya-Qiong Li, Xin-Yue Chen, Hui-Qiang Tian, Shi-Long Yin.

**Funding acquisition:** Guo Liu.

**Investigation:** Wei Dong, Qi-Xiang Miao, Wen-Bo Qiao.

**Methodology:** Guo Liu, Ye-Hui Wang.

**Project administration:** Wei Li.

**Software:** Ye-Hui Wang.

**Supervision:** Ting Zhang.

**Writing – original draft:** Guo Liu.

**Writing – review & editing:** Ye-Hui Wang.

## References

[1] Long MT Noureddin M Lim JK. AGA clinical practice update: diagnosis and management of nonalcoholic fatty liver disease in lean individuals: expert review. Gastroenterology. 2022;163:764–74.e1. 35842345 10.1053/j.gastro.2022.06.023 PMC9398982

[2] Grgurevic I Podrug K Mikolasevic I. Natural history of nonalcoholic fatty liver disease: implications for clinical practice and an individualized approach. Can J Gastroenterol Hepatol. 2020;2020:1–10. 10.1155/2020/9181368 PMC6995480 32051820

[3] Devarbhavi H Asrani SK Arab JP. Global burden of liver disease: 2023 update. J Hepatol. 2023;79:516–37. 36990226 10.1016/j.jhep.2023.03.017

[4] Ding C Fu X Zhou Y. Disease burden of liver cancer in China from 1997 to 2016: an observational study based on the Global Burden of Diseases. BMJ Open. 2019;9:e025613. 10.1136/bmjopen-2018-025613 PMC6500226 31015269

[5] Rey-Bedon C Banik P Gokaltun A. CYP450 drug inducibility in NAFLD via an in vitro hepatic model: understanding drug-drug interactions in the fatty liver. Biomed Pharmacother. 2022;146:112377. 35062050 10.1016/j.biopha.2021.112377 PMC8792443

[6] Klionsky DJ Petroni G Amaravadi RK. Autophagy in major human diseases. EMBO J. 2021;40:e108863. 34459017 10.15252/embj.2021108863 PMC8488577

[7] Debnath J Gammoh N Ryan KM. Autophagy and autophagy-related pathways in cancer. Nat Rev Mol Cell Biol. 2023;24:560–75. 36864290 10.1038/s41580-023-00585-z PMC9980873

[8] Singh R Kaushik S Wang Y. Autophagy regulates lipid metabolism. Nature. 2009;458:1131–5. 19339967 10.1038/nature07976 PMC2676208

[9] Zhang S Peng X Yang S. The regulation, function, and role of lipophagy, a form of selective autophagy, in metabolic disorders. Cell Death Dis. 2022;13:132. 35136038 10.1038/s41419-022-04593-3 PMC8825858

[10] Shin DW. Lipophagy: molecular mechanisms and implications in metabolic disorders. Mol Cells. 2020;43:686–93. 32624503 10.14348/molcells.2020.0046 PMC7468585

[11] Zhang J Feng Q. Pharmacological effects and molecular protective mechanisms of Astragalus polysaccharides on nonalcoholic fatty liver disease. Front Pharmacol. 2022;13:854674. 35308224 10.3389/fphar.2022.854674 PMC8929346

[12] Zhu Y Chai Y Xiao G. Astragalus and its formulas as a therapeutic option for fibrotic diseases: pharmacology and mechanisms. Front Pharmacol. 2022;13:1040350. 36408254 10.3389/fphar.2022.1040350 PMC9669388

[13] Liu YT Lv WL. Research progress in *Astragalus membranaceus* and its active components on immune responses in liver fibrosis. Chin J Integr Med. 2020;26:794–800. 31502184 10.1007/s11655-019-3039-1

[14] Zhou B Zhou DL Wei XH. Astragaloside IV attenuates free fatty acid-induced ER stress and lipid accumulation in hepatocytes via AMPK activation. Acta Pharmacol Sin. 2017;38:998–1008. 28344322 10.1038/aps.2016.175 PMC5519246

[15] Yanping D Xiaoqing D Yifan MA. Astragaloside IV plays a role in reducing radiation-induced liver inflammation in mice by inhibiting thioredoxin-interacting protein/nod-like receptor protein 3 signaling pathway. J Tradit Chin Med. 2023;43:87–94. 36639999 10.19852/j.cnki.jtcm.2023.01.008 PMC9924712

[16] Zaman Q Zhang D Reddy OS. Roles and mechanisms of Astragaloside IV in combating neuronal aging. Aging Dis. 2022;13:1845–61. 36465185 10.14336/AD.2022.0126 PMC9662284

[17] Wei X Yang X Hu F. Astragaloside IV activates AMPK to alleviate liver lipid deposition of nonalcoholic fatty liver disease in mice. Drug Eval. 2021;18:1230–4.

[18] Zhong M Yan Y Yuan H. Astragalus mongholicus polysaccharides ameliorate hepatic lipid accumulation and inflammation as well as modulate gut microbiota in NAFLD rats. Food Funct. 2022;13:7287–301. 35726797 10.1039/d2fo01009g

[19] Shan H Lin Y Yin F. Effects of astragaloside IV on glucocorticoid-induced avascular necrosis of the femoral head via regulating Akt-related pathways. Cell Prolif. 2023;56:e13485. 37186483 10.1111/cpr.13485 PMC10623974

[20] Xu Z Han X Ou D. Targeting PI3K/AKT/mTOR-mediated autophagy for tumor therapy. Appl Microbiol Biotechnol. 2020;104:575–87. 31832711 10.1007/s00253-019-10257-8

[21] Harikrishnan H Jantan I Haque Md A. Anti-inflammatory effects of hypophyllanthin and niranthin through downregulation of NF-κB/MAPKs/PI3K-Akt signaling pathways. Inflammation. 2018;41:984–95. 29427163 10.1007/s10753-018-0752-4

[22] Wang Y-H Zhou Y Gao X. Duhuo Jisheng decoction regulates intracellular zinc homeostasis by enhancing autophagy via PTEN/Akt/mTOR pathway to improve knee cartilage degeneration. PLoS One. 2024;19:e0290925. 38166086 10.1371/journal.pone.0290925 PMC10760926

[23] Feng H Zhu X Tang Y. Astragaloside IV ameliorates diabetic nephropathy in db/db mice by inhibiting NLRP3 inflammasome-mediated inflammation. Int J Mol Med. 2021;48:164. 34278447 10.3892/ijmm.2021.4996 PMC8262660

[24] Eslam M Newsome PN Sarin SK. A new definition for metabolic dysfunction-associated fatty liver disease: an international expert consensus statement. J Hepatol. 2020;73:202–9. 32278004 10.1016/j.jhep.2020.03.039

[25] Liu Z Wei R Li Y. Coronary heart disease is associated with nonalcoholic fatty liver disease in patients without hypertension and diabetes. Medicine (Baltim). 2020;99:e20898. 10.1097/MD.0000000000020898 PMC7328925 32590801

[26] Song Y Dang Y Wang P. CHD is associated with higher grades of NAFLD predicted by liver stiffness. J Clin Gastroenterol. 2020;54:271–7. 31305280 10.1097/MCG.0000000000001238 PMC7012352

[27] Chalasani N Younossi Z Lavine JE. The diagnosis and management of nonalcoholic fatty liver disease: practice guidance from the American Association for the Study of Liver Diseases. Hepatology. 2018;67:328–57. 28714183 10.1002/hep.29367

[28] Romero-Gómez M Zelber-Sagi S Trenell M. Treatment of NAFLD with diet, physical activity and exercise. J Hepatol. 2017;67:829–46. 28545937 10.1016/j.jhep.2017.05.016

[29] Fu J Wang Z Huang L. Review of the botanical characteristics, phytochemistry, and pharmacology of *Astragalus membranaceus* (Huangqi). Phytother Res. 2014;28:1275–83. 25087616 10.1002/ptr.5188

[30] Siying H Luo D Zhu Q. An updated meta-analysis of Chinese herbal medicine for the prevention of COVID-19 based on Western-Eastern medicine. Front Pharmacol. 2023;14:1257345. 38044944 10.3389/fphar.2023.1257345 PMC10693348

[31] Sheik A Kim K Varaprasad GL. The anti-cancerous activity of adaptogenic herb *Astragalus membranaceus*. Phytomedicine. 2021;91:153698. 34479785 10.1016/j.phymed.2021.153698

[32] Li C-X Liu Y Zhang Y-Z. Astragalus polysaccharide: a review of its immunomodulatory effect. Arch Pharm Res. 2022;45:367–89. 35713852 10.1007/s12272-022-01393-3

[33] Xie D Zhou P Liu L. Protective effect of Astragaloside IV on hepatic injury induced by iron overload. Biomed Res Int. 2019;2019:3103946. 31428632 10.1155/2019/3103946 PMC6683835

[34] Li L Huang W Wang S. Astragaloside IV attenuates acetaminophen-induced liver injuries in mice by activating the Nrf2 signaling pathway. Molecules. 2018;23:2032. 30110942 10.3390/molecules23082032 PMC6222748

[35] Grefhorst A Van De Peppel IP Larsen LE. The role of lipophagy in the development and treatment of non-alcoholic fatty liver disease. Front Endocrinol. 2020;11:601627. 10.3389/fendo.2020.601627 PMC7883485 33597924

[36] Arab HH Al-Shorbagy MY Saad MA. Activation of autophagy and suppression of apoptosis by dapagliflozin attenuates experimental inflammatory bowel disease in rats: targeting AMPK/mTOR, HMGB1/RAGE and Nrf2/HO-1 pathways. Chem Biol Interact. 2021;335:109368. 33412153 10.1016/j.cbi.2021.109368

[37] Bu H Liu D Zhang G. AMPK/mTOR/ULK1 axis-mediated pathway participates in apoptosis and autophagy induction by Oridonin in colon cancer DLD-1 cells. Onco Targets Ther. 2020;13:8533–45. 32904616 10.2147/OTT.S262022 PMC7457577

[38] Wang H Liu Y Wang D. The upstream pathway of mTOR-mediated autophagy in liver diseases. Cells. 2019;8:1597. 31835352 10.3390/cells8121597 PMC6953127

[39] Zachari M Ganley IG. The mammalian ULK1 complex and autophagy initiation. Essays Biochem. 2017;61:585–96. 29233870 10.1042/EBC20170021 PMC5869855

[40] Wang YL Zhou X Li DL. Role of the mTOR-autophagy-ER stress pathway in high fructose-induced metabolic-associated fatty liver disease. Acta Pharmacol Sin. 2022;43:10–4. 33731774 10.1038/s41401-021-00629-0 PMC8724298

[41] Kitade H Chen G Ni Y. Nonalcoholic fatty liver disease and insulin resistance: new insights and potential new treatments. Nutrients. 2017;9:387. 28420094 10.3390/nu9040387 PMC5409726

[42] Qian H Chao X Williams J. Autophagy in liver diseases: a review. Mol Aspects Med. 2021;82:100973. 34120768 10.1016/j.mam.2021.100973 PMC9585624

[43] Sapp V Gaffney L Eauclaire SF. Fructose leads to hepatic steatosis in zebrafish that is reversed by mechanistic target of rapamycin (mTOR) inhibition. Hepatology. 2014;60:1581–92. 25043405 10.1002/hep.27284

[44] Li J Fan Y Zhang Y. Resveratrol induces autophagy and apoptosis in non-small-cell lung cancer cells by activating the NGFR-AMPK-mTOR pathway. Nutrients. 2022;14:2413. 35745143 10.3390/nu14122413 PMC9228598

